# Novel Y-chromosomal microdeletions associated with non-obstructive azoospermia uncovered by high throughput sequencing of sequence-tagged sites (STSs)

**DOI:** 10.1038/srep21831

**Published:** 2016-02-24

**Authors:** Xiao Liu, Zesong Li, Zheng Su, Junjie Zhang, Honggang Li, Jun Xie, Hanshi Xu, Tao Jiang, Liya Luo, Ruifang Zhang, Xiaojing Zeng, Huaiqian Xu, Yi Huang, Lisha Mou, Jingchu Hu, Weiping Qian, Yong Zeng, Xiuqing Zhang, Chengliang Xiong, Huanming Yang, Karsten Kristiansen, Zhiming Cai, Jun Wang, Yaoting Gui

**Affiliations:** 1 BGI-Shenzhen, Shenzhen 518083, China; 2 Guangdong and Shenzhen Key Laboratory of Male Reproductive Medicine and Genetics, Institute of Urology, Peking University Shenzhen Hospital, Shenzhen PKU-HKUST Medical Center, Shenzhen 518036, China; 3 Shenzhen Key Laboratory of Genitourinary Tumor, Shenzhen Second People’s Hospital, First Affiliated Hospital of Shenzhen University, Shenzhen 518035, China; 4 Family Planning Research Institute/The Center of Reproductive Medicine, Tongji Medical College, Huazhong University of Science and Technology, Wuhan 430030, China; 5 Shool of bioscience & bioengineering, South China University of Technology, Guangzhou, China; 6 BGI-Wuhan, Wuhan, China; 7 College of Life Sciences, University of Chinese Academy of Sciences, 19A Yuquan Road, Shijingshan District, Beijing, 100094, China; 8 The Center of Reproductive Medicine, Shenzhen Zhongshan Urological Hospital, Shenzhen 518045, China; 9 Department of Biology, University of Copenhagen, Copenhagen 2200, Denmark

## Abstract

Y-chromosomal microdeletion (YCM) serves as an important genetic factor in non-obstructive azoospermia (NOA). Multiplex polymerase chain reaction (PCR) is routinely used to detect YCMs by tracing sequence-tagged sites (STSs) in the Y chromosome. Here we introduce a novel methodology in which we sequence 1,787 (post-filtering) STSs distributed across the entire male-specific Y chromosome (MSY) in parallel to uncover known and novel YCMs. We validated this approach with 766 Chinese men with NOA and 683 ethnically matched healthy individuals and detected 481 and 98 STSs that were deleted in the NOA and control group, representing a substantial portion of novel YCMs which significantly influenced the functions of spermatogenic genes. The NOA patients tended to carry more and rarer deletions that were enriched in nearby intragenic regions. Haplogroup O2* was revealed to be a protective lineage for NOA, in which the enrichment of b1/b3 deletion in haplogroup C was also observed. In summary, our work provides a new high-resolution portrait of deletions in the Y chromosome.

Male infertility affects approximately 7% of the general population, and spermatogenic failure accounts for the majority of these cases. Non-obstructive azoospermia (NOA) is a severe state of spermatogenic failure (SSF) that affects 10% of infertile men and is diagnosed in 60% of azoospermic men^1^. The etiologies of NOA are thought to include genetic disorders, such as sex-chromosome abnormalities, Y chromosomal microdeletions (YCMs) and translocations, cryptorchidism, testicular torsion, radiation and toxins^1^,^2^. YCM is the most important genetic etiology of male infertility and has been extensively studied^3^,^4^,^5^. Over the last decade, varying extents of Y chromosome microdeletions have been identified. These microdeletions are clustered in three primary regions termed AZFa, AZFb, and AZFc^6^. Common deletions sites include AZFa, AZFb, AZFc, AZFab, AZFac, AZFbc and AZFabc. Most of these recurrent deletions result from non-allelic homologous recombination (NAHR) between near-identical amplicons, including gr/gr, b1/b3, and b2/b3, which are partial deletions that occur within or near the AZFc region^7^,^8^.

Currently, the detection of Y chromosome deletions is commonly adopted for diagnostic and prognostic purposes, and is demonstrated its essentialness^9^,^10^. In clinical practice, the European Academy of Anthrology (EAA) and the European Molecular Genetics Quality Network (EMQN) have published a guideline^11^ that adopts the use of 6 sequence-tagged sites (STSs) to detect AZF complete deletions and recently have revised the guideline by adding extensional analysis on a few additional STSs^12^. Twenty to 30 STSs have been suggested to be sufficient for providing good coverage of the important regions of the Y chromosome^13^,^14^. Recently, novel functional Y chromosomal partial deletions have been recurrently reported. The majority of the studies have focused on single-plex or multiplex PCR with limited STS primers. The complex structure of the AZF region, which is composed of massive, near-perfect amplicons, poses special challenges for the sequencing of the region and subsequent characterization of the deletions that affect the region.

The emerging technique of next generation sequencing (NGS) provides a unique opportunity to depict the whole portrait of Y chromosome deletions. Whole genome sequencing (WGS), including whole Y chromosome sequencing, has enabled the tracking of Y chromosomal variations including deletions. However, the majority of deleterious deletions are dispersed along the ampliconic regions (especially in eight palindromes) that consist of a total of 5.7 Mb or 25% of the MSY euchromatin, which creates a technological difficulty for WGS because this method requires mapping based on short reads, and these regions are usually filtered for further analyses^15^. Nevertheless, focusing on only the numerous STSs within the palindrome rather than the entire sequences provides unique landmarks that can be used to track deletions. This set of STSs in combination with the NGS technique is perfectly suited for the identification of deletions across the Y chromosome.

To track the overall deletion status and prevalence across the whole Y chromosome, we collected all of the unique and low-copy number STSs of the Y chromosome in the database and designed probes to capture and sequence all of them on the NGS platform. A total of 2260 (1787 post-filtering) STSs dispersed along the Y chromosome were captured and further sequenced. We carefully recruited 766 patients (post-filtering) with NOA and excluded those with complete AZFa, AZFb or AZFc deletions (see the Methods for details) and 683 matched controls (post-filtering) with normal fertility histories from the Chinese population to test all of the STSs. In this study, we first developed a novel algorithm to detect deletions in our dataset and validated its high level of accuracy with various experimental approaches (Fig. 1). We then carefully compared the deletions and the haplogroups between the NOA and controls. Finally, we depicted the whole deletion portrait and the characteristics of our dataset. A few novel and significant Y deletions were also carefully described.

## Result

### Data production

We selected 2260 STSs that are dispersed across the entire euchromatic region of the male-specific Y chromosome (Supplementary Table 1 and Fig. 2a). Taken together, the STS sequences constituted 846,000 bp of the target region. One thousand four hundred and eighty-five Y chromosomes, including 774 from patients with NOA and 711 from healthy controls, were sequenced with Hiseq2000, and mean data amount was 25.27 Mb per sample. On average, each sample was sequenced with a mean coverage of 38.25x, and 95.86% of the target region was covered by at least one read (Supplementary Table 2).

### Method development and evaluation of the detection of  Y-chromosomal microdeletions

#### Data alignment, filtering and normalization

**Figure 1:**
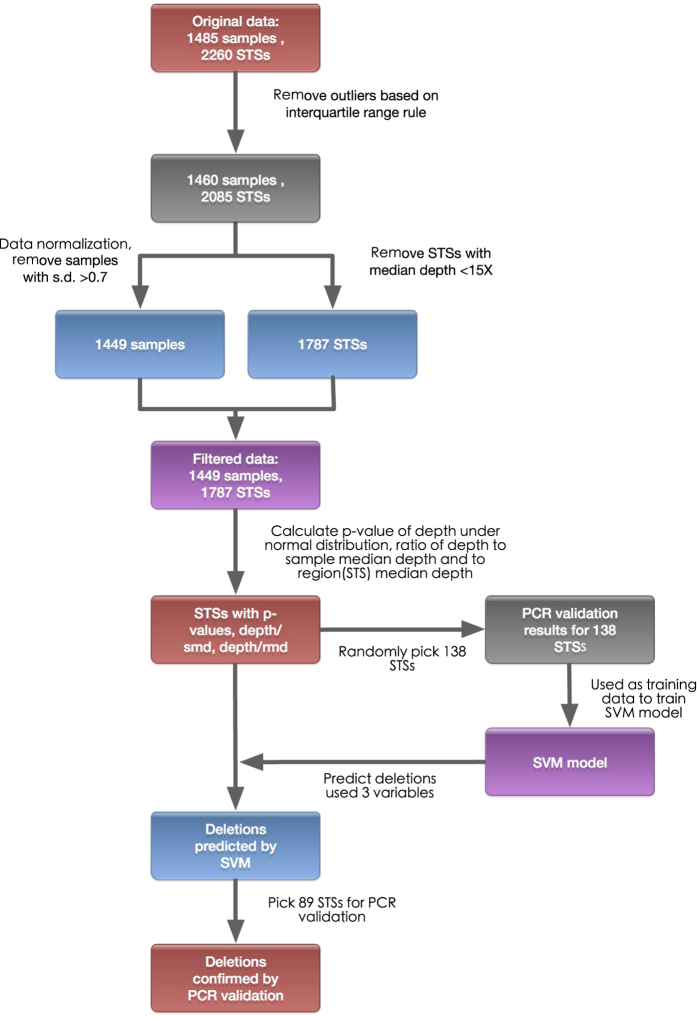
Flowchart of the detection of YCMs in this study.

Deleted STSs should have significantly lower read coverage than undeleted STSs, but the reads for the deleted STSs are not usually zero due to non-specific capture, sequencing and misalignment effects, so sequencing depth can serve as an informative signal for deletion detection. To fully utilize this information, we derived three metrics from the sequencing depth for use as predictors and developed a pipeline to detect STS deletions that utilized the support vector machine (SVM) model (Fig. 1).

For data quality control, the sequencing reads were filtered to remove low-quality and duplicated reads and were then aligned to the reference genome. The mean and median depth of each sample and STS, as well as the depth distribution of each STS and sample, were calculated. Due to the abnormal efficiency of the probes for the capture of certain STSs (GC bias effect etc.) or other issues, such as sample quality, the mean depths of certain STSs and samples deviated from the normal range; for example, extremely low STS and sample levels with depths outside of the 1.5x interquartile range were filtered as outliers to reduce the possibility of false positive detection. Twenty-five samples and 175s STS were removed in this stage (Fig. 1). Additionally, we observed that there was sufficient statistical power to qualify a STS for deletion identification only when the depth distribution of that STS was sufficiently high among all of the samples, i.e., when the STS performed well in terms of the capture of undeleted samples. After data modeling (data not shown), we set up a more stringent cutoff of 15x for the median depths of the STSs, and an additional 298 STS were filtered. The variation in the data production for each sample was normalized by dividing the depth of each STS by the mean depth of that particular sample. Furthermore, substantial depth variation across all of the STSs in one sample reflected an inefficiency of the experiment for the sample. Such samples would adversely affect the accuracy of the deletion judgment. Therefore, a filter <0.7 was applied to the standard deviations of the normalized depths, which resulted in the filtering of 11 samples; thus, 1449 samples (97.6%) and 1787 STSs (79.0%) were qualified for the next step.

#### Deletion detection

For each STS, three metrics derived from the sequencing depth were used as predictors to fit a support vector machine (SVM) model for state classification. Enlightened by the normal distribution approximation for the depth distribution of the STSs in the total sample set (excluding the outliers), we calculated the logarithm of the probability of each STS under the approximated normal distribution, which was scaled to be centered on 0 and had a standard deviation of 1; this measure provided the first metric. The other two metrics were the ratio of the mean depth of the STS to the median depth of the same STS across all samples (depth/RMD) and the ratio of the mean depth of the STS to the median depth of all STSs in that sample (depth/SMD). Details are provided in the Methods section.

To train the SVM model, a total of 134 randomly selected STSs from 26 samples that covered different p values, depth/RMD and depth/SMD values were selected to perform the PCR validation to reveal their microdeletion states and were used as a training data set for the SVM model (Supplementary table 3). In the SVM model, a Gaussian radial-based kernel were used, and its parameters were selected by a grid search with exponentially growing C and sigma. This process was performed via cross validation using the training dataset. The concordance rates for the different C and sigma are illustrated in Supplementary Fig. 1, and the best combination of C = 2^9 and sigma = 2^3 was selected. With this combination, the SVM model perfectly classified the training samples.

Next, we used the trained SVM classifier to detect the STS deletions in all of the samples. Overall, 1020 deletions in 87 NOA patients and 264 deletions in 71 normal donors were identified by our method (Table 1 and Supplementary Table 4).

#### Accuracy evaluation

To validate our results, we first examined the statuses of the two control STSs in our dataset (sY84 and sY86, see Methods), and no deletions were detected by our method in any of the samples as expected. Furthermore, 89 events that included both deletions and non-deletions from 17 STS and from 16 samples were randomly selected for the PCR validation, and a high validation rate of 97.8% (87/89) was achieved (Supplementary Table 5 and Supplementary Table 6). We also used the frequently deleted STS sY1191 to estimate our false negative detection rate. Four of one hundred random controls and 7/100 NOAs deleted by PCR were all detected in our method. Finally, we found that all of the Y chromosomes belonging to haplogroup N had a b2/b3 deletion in our dataset, and this finding is consistent with that of a previous report^8^, which implies the accuracy of our method for the detection of deletions and haplogroup clustering.

### NOA patients carry more and rarer deletions that are enriched in gene regions

**Figure 2:**
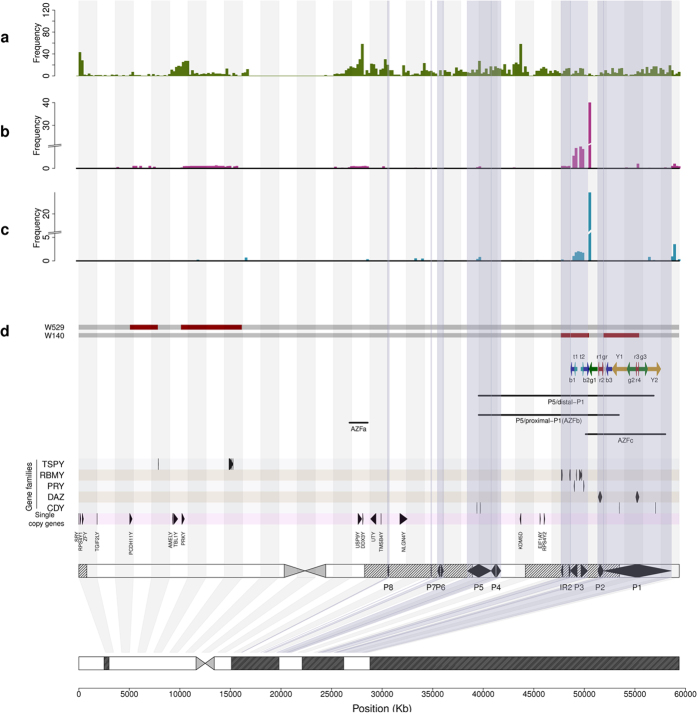
Distributions of the STS markers and deletions in the Y chromosome. (**a**) The density distribution of the STS markers targeted in our study and (**b**) the density distributions of the STS deletions in the NOA patients (**c**) and the normal group across the Y chromosome. (**d**) Illustrations of novel deletions from samples w529 and w140. The densities were calculated for every 100 kb window.

**Figure 3:**
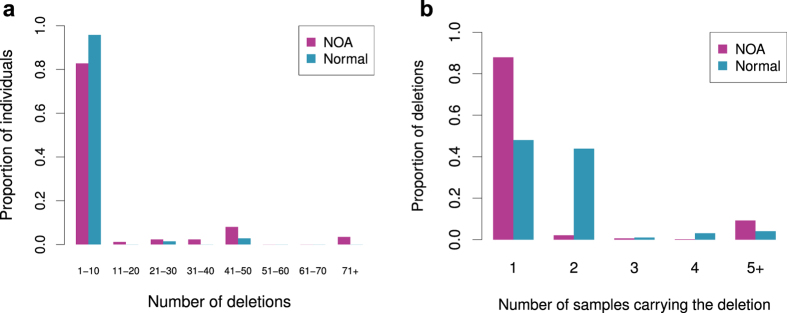
NOA patients carry more but rarer deletions. (**a**) The non-cumulative distributions of the STS deletion frequencies in the NOA patients and controls. The X-axis indicates the number of deleted STS found in each individual, and the Y-axis indicates the proportions of individuals who carried the each number of STS deletions in that window. The red and blue bars indicate the NOA patients and the normal group, respectively. (**b**) The recurrence of the STSs that were deleted in each group. The X-axis indicates the number of individuals with deleted STSs, and the Y-axis indicates the proportion of the total unique STSs that were deleted in the respective numbers of individuals. The red and blue bars indicated the NOA and normal groups, respectively.

**Figure 4:**
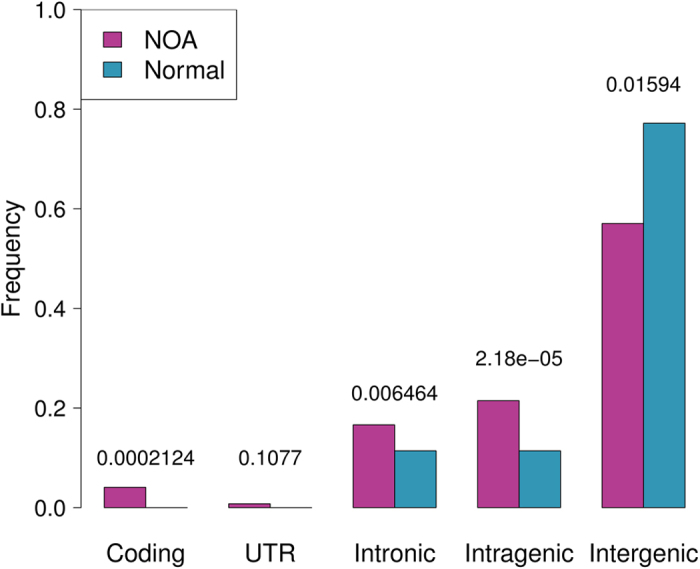
NOA deletions were enriched near intragenic regions. Intragenic regions are a combination of coding, UTR and intronic region, and the *p* values were calculated by Chi square tests, which were listed on top of the bars.

Overall, 87 NOA patients were identified as having at least one deleted STS, and these patients constituted 11.4% of the total of 766 cases. In contrast, this number was lower in the control group (71/683, 10.4%), but the difference was not significant (P = 0.5797, table 1). Comparison of the total deleted STSs in the NOA and control groups revealed that significantly more STSs were deleted in the NOAs than in the controls (1020 vs. 264, P < 2.2E-16). The average deletion numbers of the individuals with NOA and the control group also exhibited a significant difference (11.7 vs. 3.7, P < 0.001), with a 3.2-fold increase in the NOA patients. This trend is clearly illustrated in Fig. 3a. Although the majorities of the individuals in both groups had fewer than 5 deleted STSs, the distribution curves were obviously inclined toward a larger number of deletions in the case group than the controls. Three NOA patients had more than 75 STS deletions, while none of the control samples had similar numbers of deletions. Interestingly, in addition to the hotspots in AZF regions that were present in both the case and control groups, the deleted STSs of the cases tended to be aggregated, while those in the controls tended to be dispersed (Fig. 2b,c). After connecting the deleted STSs (see Methods), the unique deletions in the NOAs were significantly reduced from 481 to 121, whereas no significant shrinkage was observed in the controls (98 to 86). These findings demonstrated the aggregated nature of the deletions in the NOA group. Furthermore, these deleted STSs were significantly enriched near the coding sequences (defined as sharing an overlapping base pair, p < 0.001) and the genes (p < 0.0001) in the NOA group compared with the controls. The deletions in the controls tended to occur in intergenic regions (P < 0.05). Indeed, we found that none of the deleted STSs in the controls was located in the coding or UTR regions on the Y chromosome (Fig. 4 and Supplementary Table 7). In summary, NOA patients typically carry large deletions that constitute numerous continuous STSs and would induce the loss of functional genes or gene copies, whereas the deletions in the controls tended to be short deletions that involved fewer STSs and functional genes.

The NOA and control groups exhibited 481 and 98 unique STS deletions, respectively, and the difference was significant (P < 2.2 × 10^−16^, chi Square test). Examination of the recurrences or frequencies of these STS revealed that the average number of NOAs who carried each unique deletion was 2.1, and this value was significantly lower than the average of 2.7 per deletion observed in the control group (P = 7.4 × 10^−16^, Kruskal-Wallis test, Table 1). The frequency distribution of each deleted STS revealed that more than 80% of the deletions in the NOA group were unique and only occurred in one individual (Fig. 3b). Further analysis confirmed that these unique deletions represented several long-range deletions with functional importance. In contrast, in the control group, more than half of the deletions were recurrent and scattered in the Y chromosome. Interestingly, the NOAs carried more recurrent deletions with high frequencies (harbored by more than 5 samples) than the controls, and a detailed investigation revealed that these deletions were located in the AZFc region (*DAZ* gene deletion). This issue is discussed in detail below.

Our STS markers were spread across all of the ampliconic regions of the Y chromosome and were intensively colonized with Yp and the large palindromic segments from P1 to P8 that spanned the azoospermia factor (AZF) genes (Fig. 2a). The prevalence of deletions across the Y chromosome revealed specific patterns in the NOA and control samples (Fig. 2b,c). First, AZFc harbored several recurrent interstitial deletions, and their frequencies in the NOA group were higher than those of the control group. Second, there were some long-range continuous deletions that were in Yp, but this only occurred in the NOAs. Gene analysis revealed that these deletions were enriched in the gene-rich regions and that the majority of these deletions influenced specific single-copy genes or gene copies of specific gene families. Moreover, some of these deletions might even have caused the loss of the functions of all of the gene copies. Gene copies, including *TSPY* in Yp, *RBMY* in AZFb and *DAZ* in AZFc (Table 2), were lost. In contrast, the deleted STSs in the controls were more likely to be located in the gene desert of the Y chromosome.

### Known and novel deletions and their relationships with NOA

**Figure 5:**
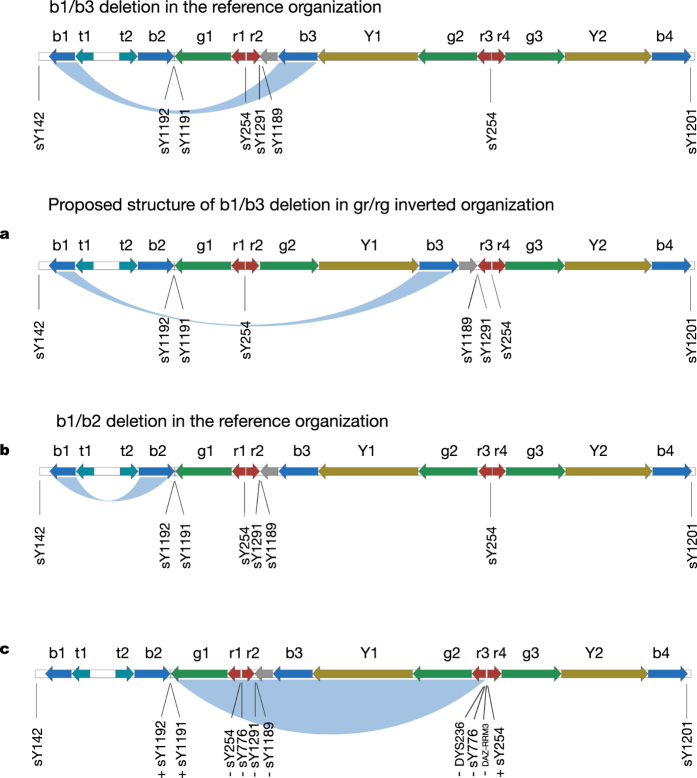
Illustration of the novel deletions. (**a**) Illustration of the b1/b3 deletion in the reference organization and the proposed structure of the b1/b3 deletion with an gr/rg inverted organization. (**b**) Illustration of the b1/b2 deletion in the reference organization. (**c**) Illustration of a *DAZ* deletion. The deletion statuses of the STS markers are marked, and “+” indicates “not deleted”, and “−” indicates “deleted”.

In our study, the deletions were detected with no prior knowledge. After connecting the deleted STSs, we were able to identify a substantial portion of the known/published YCMs, and the majority of these YCMs were in AZF regions (with the exception of gr/gr for which the only marker STSs, i.e., sY1291 and sY1189, were filtered in our dataset). To confirm the reliability of our categorization of the AZF partial deletions, particularly the most abundant AZFc deletions, we performed PCR for sY1191, sY1192, sY1291 and sY1189 to differentiate the partial AZFc deletions^5^, and the results were fully consistent with the sequencing classification. In our NOA samples, the most abundant AZFc deletions were b2/b3 (7.0%) followed by b1/b3 (0.91%). In addition to the known YCMs, two small deletions were identified in AZFa and AZFb (Table 2 and Supplementary Table 4). These deletions influenced none of the three major genes, i.e., *USP9Y*, *DBY* (*DDX3Y*) and *UTY,* in the AZFa region and only influenced the partial copies of *CDY* in the AZFb region. These deletions were found in both the NOAs and controls and were most likely polymorphic and not involved in male reproduction. Interestingly, we found two novel forms of AZFc partial deletion. The first was carried by two NOAs and deleted all the STSs from b1 to b2 along with sY1191 and sY1192, whereas the deletion retained sY1291 and sY1189. It is most likely that this unusual b1/b3 deletion arose from a gr/rg inversion (Table 2 and Fig. 5a). The other novel deletion was a b1/b2 deletion (Table 2 and Fig. 5b) that was carried by two of the NOA patients (0.26%), and one of these patients had Sertoli-cell-only syndrome. This deletion was further validated by PCR. The b1/b2 deletion caused a partial loss of the *RBMY* gene copies and the complete loss of the *PRY* copies, which might impact spermatogenesis. Additionally, we identified a novel form of an entire *DAZ* gene deletion that would have been falsely defined as a gr/gr deletion with limited STS PCR (Table 2 and Fig. 5c). Five NOA patients (0.65%) were absent all of the *DAZ* gene copies, while the majority of the nearby genes were unaffected.

Two novel, massive deletions were specifically detected in the NOA patients and merit particular attention. Both patients had the normal karyotype of 46, XY. The patient with w529 had a testicular volume of 15 ml, and the testicular biopsy and histological analysis revealed that the development of his sperm cells was arrested in the spermatocyte stage. We identified two massive deletions separated by a distance of 1.1 Mb and with a total size of approximately 3.37–3.86 Mb in the Yp chromosome of this patient (Fig. 2d and Table 2). These deletions affected the functions of several protein-coding genes, including *PCDH11Y, PRKY* and *TSPY.* Notably *TSPY* is thought to function in early spermatogenesis and to be involved in the differentiation and proliferation of the spermatogonia-spermatocyte transition^16^. Patient w140, who had a soft testis with the small size of 6 ml, exhibited a discrete deletion from the proximal IR2 that included the b1/b2 and gr/gr regions (Fig. 2d and Table 2). This significant deletion spanned approximately 2.5 Mb and disrupted all of the genes copies of *RBMY* and *PRY* and the partial copies of *DAZ* and *CDY*. The *RBMY* gene in the AZFb region has long been considered to be vitally functional in spermatogenesis and reported to be deleted in a couple of patients with spermatogenic failure^17^. The deletion of this gene in combination with a gr/gr deletion which is a risk factor for spermatogenic failure^5^, might have induced this severe case. The deleted STSs in all of the samples are listed in Supplementary Table 4.

### Y haplogroups and deletions

Several studies have reported associations of certain Y haplogroups with male infertility^18^,^19^,^20^,^21^, while others have opposed this this observation^22^. With the help of our substantial sequencing coverage and the ability to capture the flanking sequences of the target STSs, we were able to call SNPs in the Y chromosome, including substantial markers, to determine the Y haplogroups. We attempted to assign our samples to haplogroups based on updated markers to differentiate the Y haplogroup tree linage^23^ and were able to assign 589 NOA patients and 569 normal controls into 9 major Y haplogroups and 5 additional sub-lineages in haplogroup O (Table 3). Complete information for the marker SNPs in the remaining samples were not confidently recovered and were thus excluded from further analysis. The majority of our samples were spread among the haplogroup O, C, N and Q, and the greatest proportion belonged to haplogroup O (74.4% among the NOAs and 79.1% among the normal controls), supporting the high prevalence of this haplogroup in East Asia. Association analysis revealed that haplogroup O2* seemed to be a lineage that was protective against NOA (Table 3, P = 1.6 × 10^−3^, Fisher’s exact test), while the O1a2 haplogroup was only marginally associated to be susceptible (P = 0.035). In light of the discoveries of the enrichment of certain YCMs in certain Y haplogroups due to founder mutations that led to deletions, such as that of b2/b3 in haplogroup N and gr/gr in haplogroup D2b^7^, we intended to identify new association between the haplogroups and the deletions. Heatmaps were drawn for the STS deletion distributions of each haplogroup separately for the NOA and normal cohorts (Supplementary Fig. 2 and Supplementary Fig. 3). The observed deletion numbers were tested to determine whether they were significantly over-represented or under-represented relative to the expected numbers. The three STS deletions DYF155S1, RH102047 and sY1191 were part of the b2/b3 deletion, which was significantly enriched in haplogroup N among both the NOAs and the controls, as reported previously. Interestingly, we identified a few STS deletions that were significantly enriched in haplogroup C among the NOAs, and all of these STSs actually represented b1/b3 deletions, which implies the existence of an unidentified sub-lineage in haplogroup C that might feature the b1/b3 deletion. The exact deletion numbers and the calculated P value are listed in Supplementary Table 8 and 9.

## Discussion

Our work represents the first study to utilize next generation sequencing (NGS) of a high density of STS markers to fine map the high polymorphic deletions/microdeletions across male-specific Y chromosome (MSY) in both non-obstructive azoospermic and fertile populations. From FISH to multiplex/single-plex STS-based PCR, numerous methods have been proposed for the detection of Y chromosome deletions, including real time PCR^24^ and array CGH^25^. The key focus of these method developments is improving sensitivity and resolution. In a clinical setting, including more STS markers may not be justified in terms of cost and the relevance to clinical interpretation. Nevertheless, increasing the resolution of the current understanding of YCMs in different population, particularly the rare/partial deletions that are associated with spermatogenic failure, is highly attractive. Furthermore, a limited number of STS markers would occasionally induce false positive detections in cases in which the SNP exists in the primer annealing position^26^, but the addition of more markers in the same region would decrease this possibility. Our method combines the low cost/high throughput of the NGS technique with easy to use of STS markers to depict the most comprehensive and highest resolution landscape of YCMs to date, which significantly enhances our understanding of the field. Our method involves the sequencing of less than 1 million target bases but compromises almost two thousand STSs and thus balances resolution and cost. On one hand, compared with the traditional PCR-based approach with limited STS markers, our method not only significantly increases the resolution to allow for precise detection of the boundaries of the deletions but also provides nucleotide information for short-variation detection, which helps to assign Y haplogroups. On the other hand, compared with the whole genome or the Y chromosome sequencing, our method not only removes the difficulty of short-read alignments to repetitive and palindromic regions to allow for the detection of deletions within these regions but also greatly reduces the cost. The cost of sequencing with our method is just 1/50 of that of whole exome sequencing and 1/1000 that of whole genome sequencing. In summary, we have provided a realistic method for profiling high-resolution YCMs at the population level.

Cases with complete AZFa, AZFb or AZFc deletions, which are known to have significant clinical implication in spermatogenic failure, were excluded from our study. The aim of our study was to uncover novel deletions inside and outside of the AZF regions, including partial AZF deletions in the population and particularly those that may be involved in the genetics of NOA. Previous study has reported novel partial AZFc deletions other than b1/b3, b2/b3 and gr/gr in men with azoospermia or severe oligozoospermia, and some of them may be associated with sperm count^27^. The large sample size and fine resolution of our data did provide a population-scale portrait of YCMs and identify substantial novel NOA-specific YCMs. Some of these YCMs, such as b1/b2 and DAZ deletion, couldn’t arise from homology-mediated recombination, but are more likely to result from ligation of DNA break ends through non-homologous end joining or microhomology-mediated end joining DNA repair pathways^27^,^28^,^29^. The frequency of complete AZF deletion in infertile men (azoospermia and oligospermia) is approximately 10% in the East Asian population^12^, although the corresponding figure is not available for NOA. Excluding complete AZF deletions, the frequency of NOA with deletions was 11.4% in our data; therefore, we speculate that the total frequency of NOA with any deletions should be approximately 20%. This figure indicates a high prevalence of YCMs as genetic etiologies of NOA. Strikingly, we reported that 10.4% of fertile men had at least one STS deleted in the Y chromosome. This number is significantly higher than that previously reported and expected, and this difference is clearly attributable to our adoption of extensive STS markers. Although our analysis revealed that the deletions in fertile men usually involved fewer and more recurrent STSs, and few of these deletions directly removed genes or gene copies, considering that these deletions span at least several hundred base pairs, this extraordinary phenomenon reflects the fragility of the Y chromosome. The deletions found in the fertile population may be considered frequent polymorphisms and could also be risk factors for other diseases, such as cancer, with higher rates of mortality^30^. Such deletions could also induce functional deletions in the next generation as has been reported for partial AZFc deletions, which are a risk for complete AZFc deletion^31^. The deletions in the NOA patients reflect much more functional significance and the involvement of gene regions. As stated previously, more than 80% of the deletions in the NOAs were unique. An unanswered question is thus whether these deletions were inherited from the patients’ fertile fathers and serve as rare variants/polymorphisms that are involved in the dysfunction of male spermatogenesis with very high penetrance or whether they occurred as de novo deletions. Unfortunately, we were not able to access the genetic material of the fathers of the patients to clarify this issue. Considering the rarity of novel NOA-related deletions, the samples size should be further increased to uncover many more discoveries.

The frequencies and genetic risks of common AZFc deletions related to spermatogenic failure have been extensively studied^5^,^31^,^32^,^33^. In our NOA sample, the most abundant AZFc deletions were b2/b3 (7.0%) followed by b1/b3 (0.91%) and b1/b2 (0.26%). The frequency of the b2/b3 deletion varies between different populations, but nearly every individual in haplogroup N was determined to carry this deletion^8^. The high frequency of the b2/b3 deletion in our study was mostly due to the prevalence of haplogroup N. Excluding these samples dramatically reduced the frequency of the b2/b3 deletion to 1.2% in both the NOA patients and normal controls, which is consistent with the results of a previous report^5^. The effect of the b2/b3 deletion on spermatogenic failure remains controversial^29^, and some groups have found that b2/b3 deletion is a risk factor and is associated with spermatogenic failure^32^,^34^. In our study, although the frequency of the b2/b3 deletion was higher in the NOA patients than the normal controls (7.0% vs. 6.1%), no significant difference was observed (P > 0.2, chi square test), and the exclusion of haplogroup N provided a similar conclusion (1.2% vs. 1.2%). Our result supports the conclusions of a previous large-scale study^5^.

Our deletion picture consists of many novel deletions with clinical implications and etiological mechanisms that remain to be investigated. Specifically, regarding the deletions only observed in the NOA patients, it was necessary to include the related STSs in the panel to screen the large cohort for spermatogenic failure. We believe certain of the deletions are recurrent with high clinical penetrance. Our database could serve as a valuable resource for future investigations into deleterious YCMs, and this database should be further expanded by sequencing more infertile men and more general population subjects with our method.

## Methods

### Samples selection

All of the peripheral blood samples were collected from the Peking University Shenzhen Hospital and the Center of Reproductive Medicine, Tongji Medical College, Huazhong University of Science and Technology. NOA patients were only recruited to the study if they met the following criteria: no sperm detected in the pellets of semen samples taken on three different occasions; no inflammation or injury of the reproductive system or pelvic cavity; and no karyotypic abnormality or known Y-chromosomal microdeletion. The Y-chromosomal microdeletions were detected as described previously^11^. Specifically, sY84 and sY86 for were examined in the AZFa region and sY127 and sY134 were examined in the AZFb region and combined with sY254 and sY255 in the AZFc region, the results were used to screen for complete deletions involving AZFa, AZFb and AZFc. Testicular biopsy and histological analysis were conducted for the azoospermic men whenever possible. All of the control men had fathered at least one child without assisted reproductive techniques, such as IVF, ICSI and IMSI. This study was approved by the ethical committees of Peking University Shenzhen Hospital and Tongji Medical College, and all participants signed a consent form permitting the collection and use of their blood samples in the study. All experiments were preformed in accordance with the approved guidelines and regulations.

### STS selection and probe design

Two thousand three hundred fourteen STSs, including 2029 single-copy STSs and 285 two-copy STSs were extracted from the UniSTS databases (NCBI MapView). ,Two thousand three hundred and seventy six STSs including 2026 single-copy STSs and 350 multi-copy STSs were extracted from the UCSC database. In total, 2657 non-redundant different STSs from these two sources were selected to blot against an hg19 reference. The STSs with alignments exceeding 95% identity and 95% coverage were selected, and these STSs included 1657 STSs with unique mapping locations that constituted a net length of 467,535 bp and 603 STS with multiple alignment positions (selected from only the AZF region) that constituted 193,911 bp. Overall, 2260 STSs with a net length of 660,598 bp were ultimately selected for probe design, and there was a high density of 1063 STSs in the AZF region. The probes were designed following the standard pipeline of Nimblegen (Roche Nimblegen Inc., USA) with adjustments of the parameters to recover the majority of the target STSs.

### Library construction and high throughput sequencing for Y-chromosomal STSs

Genomic DNA was extracted from the peripheral blood with a commercial kit and was then fragmented to 200 bp by Covaris S2 (Covaris Inc.), end repaired, A-tailed and adaptor ligated. The product was purified with Ampure beads and amplified with 6 cycles of ligation-mediated PCR. Sequence capture was performed according to the manufacturer’s protocol (Roche Nimblegen Inc.), and the enriched product was further amplified by PCR. The sequencing libraries were then subjected to quality assessment, quantification, and cluster generation and then sequenced on the Hiseq 2000 platform (Illumina Inc.) with 100-bp paired-end protocols.

### Data alignment, filtering and deletion detection

The sequencing reads were first processed to remove the sequencing adaptors and discard the low quality reads and were then were aligned to hg19 of the human reference genome with SOAP2 version 2.20. PCR and optical duplicate reads were removed, and the mean and median depths of each target STS were then calculated. The samples and STSs with extreme depths were defined as outliers according to a 1.5 × IQR rule (i.e., an interquartile range rule) and were removed. Additionally, we determined that there was sufficient statistical power to qualify a STS for deletion identification only if its depth distribution was sufficiently high among all of the samples; in other words, the method performed well in the capture of undeleted samples. After data modeling (data not shown), we applied a more stringent cutoff of 15x for the median depths of the STSs. The depth of each sample was normalized by dividing it by the mean depth of all of its STSs. For each sample, the standard deviation of the normalized depth was calculated across all of the sample’s STSs, and samples with standard deviations >0.7 were excluded.

For m STS in n samples, 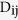 represented the mean depth of all of the bases of the jth STS in the ith sample; thus,


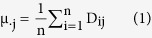



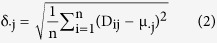


where 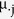 and 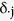 are the mean and standard deviation of the depth of the jth STS, respectively. Therefore, the probability of 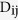 under an approximated normal distribution of the depth of the jth STS was as follows:


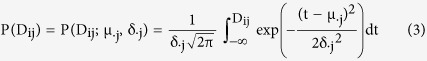


We used 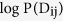 as our first predictor in the SVM model. For the second predictor, we had the following:


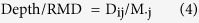


where 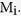 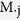 is the median of 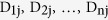, which were the depths of the jth STS among all of the samples. Similarly, we used


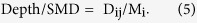


as our third predictor. 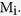 is the median of 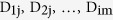.

Thus, the SVM model


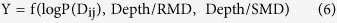


was trained and used for deletion prediction. The best combination of C and sigma parameters was selected with a grid search with exponentially growing C and sigma sequences (i.e., 2^−5^, 2^−4^, 2^−3^, .. 2^15^; Supplementary Fig. 3). The concordance rates of the different combinations of parameters were calculated via 1000 repetition 4-fold cross validation using the PCR-validated data from 134 STSs (Table S).

### Merge of the STS deletions to locate the boundaries

We attempted to merge the continuous STS deletions to locate the deletion boundaries. The principle was to merge the continuous unique deleted STSs and the undeleted multi-copy STSs for which the inter-distance was within a certain range, and there were no non-deleted single-copy STS in the merged products. To achieve this goal, we first evaluated the inter-distance distribution of the STS probes (Supplementary Fig. 4) and found that the peak was shorter than 5 kb, and the 95^th^ percentile of the distance was approximately 50 kb. To balance the possibilities of disconnecting continuous deletions and connecting discontinuous deletions, we set 100 kb as the cutoff for connecting the deleted STSs. Therefore, the continuously deleted STSs with less than 100 kb in distance were connected to represent longer deletions that composed all of the STSs. Discontinuously deleted STSs of less than 100 kb with only non-deleted multi-copy STSs between them were also connected because the multi-copy STSs might lose copies that our method was not intended to detect. The connections were applied, the deletion boundaries were inferred from the reference genome, and the approximated boundaries were estimated based on the genomic coordinates of the deleted STSs at the borders of the connections.

### PCR validation

The characterizations of the Y-chromosome microdeleted patients were validated by amplifying the STS markers with a male control sample, a female sample, and a blank sample. The STSs were sY3127, sY1241, sY1783, sY82, sY1180, sY84, sY709, sY1066, sY744, sY1264, sY1227, sY1302, sY143, sy1258, sY1259, sY1161, sY1160, sY1058, sY1616, sY1197, sY1161, sY1192, sY1191, sY1189, sY1291, and sY1206. Additionally, 100 NOA patients and 100 normal controls were randomly selected for validation using sY1191-, sY1192-, sY1189-, and sY1291-specific primers. All of the PCR assays were performed in a total volume of 25 μl that contained 100 ng of each DNA sample with the primers for the SRY gene as positive controls. The cycling protocol was as follows: 5 min at 94 °C, followed by 35 cycles as 94 °C for 45 s, 55–62 °C for 45–60 s and 60 s at 72 °C, and 72 °C for 5 min. The PCR products were analyzed by electrophoresis at 100 V on 2% agarose gels.

## Additional Information

**Accession codes**: The sequencing data have been deposited in the NCBI Sequence Read Archive (SRA) under the accession number SRA237673.

**How to cite this article**: Liu, X. *et al.* Novel Y-chromosomal microdeletions associated with non-obstructive azoospermia uncovered by high throughput sequencing of sequence-tagged sites (STSs). *Sci. Rep.* **6**, 21831; doi: 10.1038/srep21831 (2016).

## Supplementary Material

Supplementary Information

Supplementary Dataset 2

## Figures and Tables

**Table 1:** Statistics for the deletions in the NOA patients and normal individuals.

	NOA	Normal	*P* value
Number of samples	766	683	NA
no deletion	679(88.6%)	612(89.6%)	NA
any deletion	87(11.4%)	71(10.4%)	5.8 × 10^−1^*
unique deletion number	481	98	<2.2 × 10^−16^*
post-merge unique deletion	121	86	NA
Average unique deletion per deleted individual	5.5	1.4	NA
total deletion number	1,020	264	<2.2 × 10^−16^*
Average individuals per unique deletion (SD)	2.1(4.8)	2.7(6.4)	7.4 × 10^−16^**
average deletion number per deleted individuals (SD)	11.7(27.2)	3.7(7.1)	6.4 × 10^−4^**

^*^Chi square test.

^**^Kruskal-Wallis test. NA indicates not applicable.

**Table 2:** Known and novel deletions identified in our study.

	Region	AZFa***,†			AZFc				Yp†
			AZFb****,†		b2/b3	b1/b3	b1/b2†	DAZ deletion†	
NOA	frequency	2(0.26%)	1(0.13%)	1(0.13%)	54(7.0%)	7(0.91%)**	2(0.26%)	5(0.65%)	1(0.13%)
	sample ID	W606, W635	W600	W140	*	W306,W344,W135,W047.W241,W451,W688	W563,W216	W074,W1404,W141,W315, W461	W529
Normal	frequency	1(0.15%)	1(0.15%)	0	42(6.1%)	2(0.29%)	0	0	0
	sample ID	1074	247		*	1871,1973			
Major genes affected			*CDY*	*RBMY, PRY, DAZ, CDY*	*DAZ, CDY*	*RBMY, PRY, DAZ*	*RBMY, PRY*	*DAZ*	*TSPY, RBMY, PCDH11YPRKY*

^*^Details not indicated.

^**^Includes two b1/b3 deletions (W451 and W688) with the gr/rg inverted organization.

^***^Indicates that various forms of partial deletions occurred within the AZFa or AZFb regions.

^†^Novel deletions

**Table 3:** Y haplogroup distributions of the NOA patients and the normal controls.

Y Haplogroup	sub-lineage	NOA	Normal	*P* value*
J		2(0.3%)	2(0.3%)	1
T		1(0.1%)	0	1
N		45(7.6%)	34(6.0%)	2.9 × 10^−1^
Q		28(4.8%)	16(2.8%)	9.2 × 10^−2^
C		63(10.7%)	47(8.3%)	1.6 × 10^−1^
D		10(1.7%)	14(2.5%)	4.1 × 10^−1^
R		2(0.3%)	5(0.9%)	2.8 × 10^−1^
G		0	1(0.2%)	4.9 × 10^−1^
O	O2*	0	9(1.6%)	**1.6** × **10**^**−3**^
	O3a	394(66.9%)	388(68.2%)	6.6 × 10^−1^
	O2a	41(7.0%)	42(7.4%)	8.2 × 10^−1^
	O2b	2(0.3%)	4(0.7%)	4.4 × 10^−1^
	O1a2	1(0.2%)	7(1.2%)	3.5 × 10^−2^
total		589	569	

^*^Fisher’s exact test, one sided.
