# Supplementary material for: Novel Y-chromosomal microdeletions associated with non-obstructive azoospermia uncovered by high throughput sequencing of sequence-tagged sites (STSs)
Source: Sci Rep. 2016 Feb 24;6:21831. doi: 10.1038/srep21831 (PMC4764820; doi:10.1038/srep21831)
Supplement: Supplementary Information [file srep21831-s1.doc]

# Novel Y-chromosomal microdeletions associated with non-obstructive azoospermia uncovered by high throughput sequencing of sequence-tagged sites (STSs)

Xiao Liu^1,9,#^, Zesong Li^3,#^, Zheng Su^1,#^, Junjie Zhang^5,#^, Honggang Li^4^, Jun Xie^2^, Hanshi Xu^1,7^, Tao Jiang^1^, Liya Luo^3^, Ruifang Zhang^1^, Xiaojing Zeng^1^, Huaiqian Xu^6^, Yi Huang^3^, Lisha Mou^3^, Jingchu Hu^1^, Weiping Qian^2^, Yong Zeng^8^, Xiuqing Zhang^1^, Chengliang Xiong^4^, Huanming Yang^1^, Karsten Kristiansen^9^, Zhiming Cai^3,*^, Jun Wang^1,*^, Yaoting Gui^2,*^

^1^BGI-Shenzhen, Shenzhen 518083, China; ^2^Guangdong and Shenzhen Key Laboratory of Male Reproductive Medicine and Genetics, Institute of Urology, Peking University Shenzhen Hospital, Shenzhen PKU-HKUST Medical Center, Shenzhen 518036, China; ^3^Shenzhen Key Laboratory of Genitourinary Tumor, Shenzhen Second People's Hospital, First Affiliated Hospital of Shenzhen University, Shenzhen 518035, China; ^4^Family Planning Research Institute/The Center of Reproductive Medicine, Tongji Medical College, Huazhong University of Science and Technology, Wuhan 430030, China. ^5^Shool of bioscience & bioengineering, South China University of Technology, Guangzhou, China; ^6^BGI-Wuhan, Wuhan, China; ^7^College of Life Sciences, University of Chinese Academy of Sciences, 19A Yuquan Road, Shijingshan District, Beijing, 100094, China; ^8^The Center of Reproductive Medicine, Shenzhen Zhongshan Urological Hospital, Shenzhen 518045, China; ^9^Department of Biology, University of Copenhagen, Copenhagen 2200, Denmark;

^#^These authors contributed equally to this work;

*Correspondence should be addressed to Z. C. ([caizhiming2000@163.com](mailto:caizhiming2000@163.com)), J. W. ([wangj@genomics.cn](mailto:wangj@genomics.cn)) and Y. G. (guiyaoting2007@aliyun.com)

Supplementary Figures

Supplementary Fig 1 **The selection of the C and sigma for the SVM model**

Supplementary Fig 2 **Heatmap of the enrichment of STS deletions in the different haplogroups of the NOA samples.**

The red indicates overrepresentation (observed deletions /expected>1), and the green indicates underrepresentation (observed/expected<1). The expected deletion numbers in each haplogroup followed their distributions. The STS deletions harbored by more than samples are included in the figure. * P<0.001, + P<0.01, Fisher’s exact test.

Supplementary Fig 3 **Heatmaps of the enrichments of STS deletions in the different haplogroups among the normal samples.**

The red indicates overrepresentation (observed deletions /expected>1), and the green indicates underrepresentation (observed/expected<1). The expected deletion numbers in each haplogroup followed their distributions. The STS deletions harbored in more than 4 of the samples are included in the figure. * P<0.001, Fisher’s exact test

Supplementary Fig 4 **The inter-distance distribution of the STS markers**


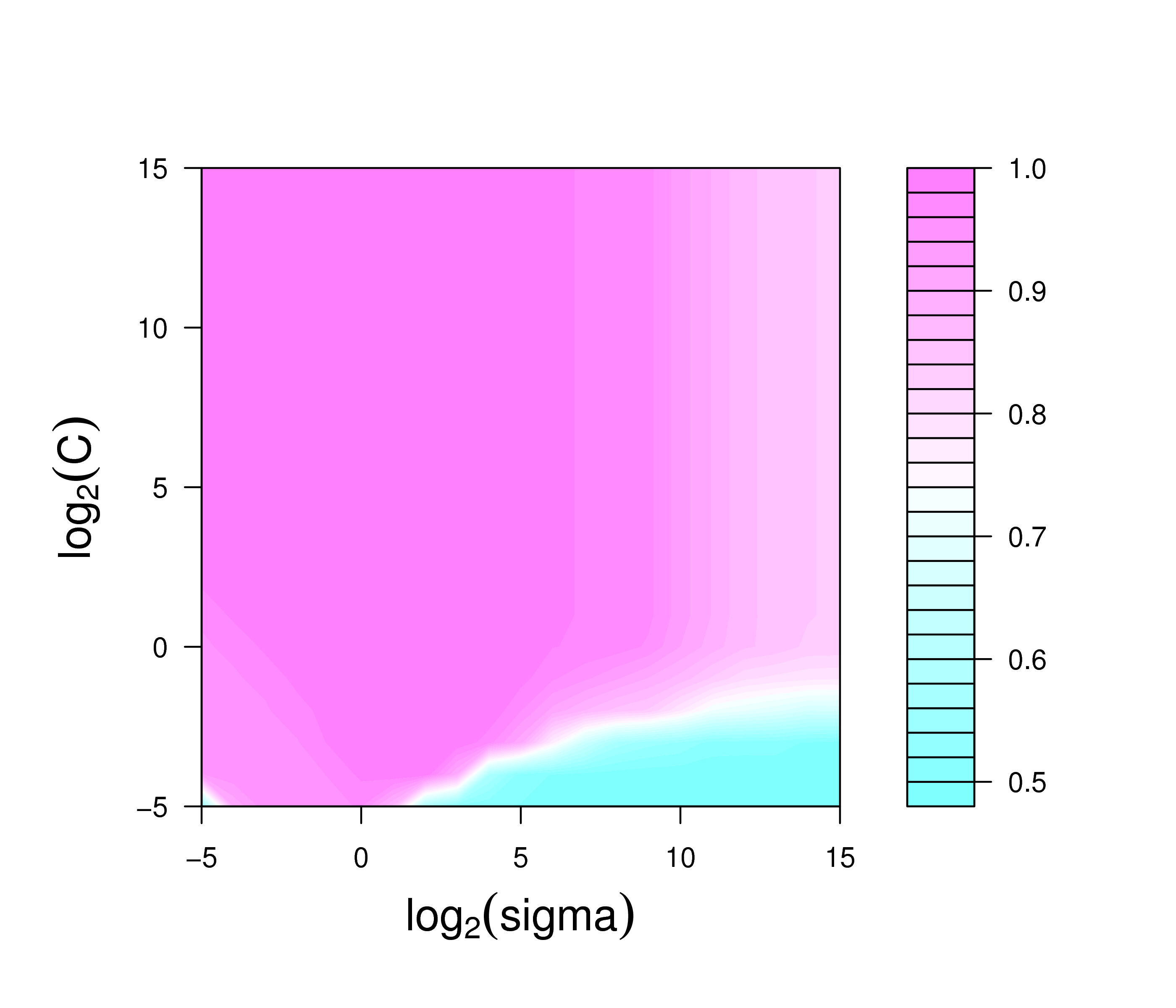


Supplementary Fig. 1


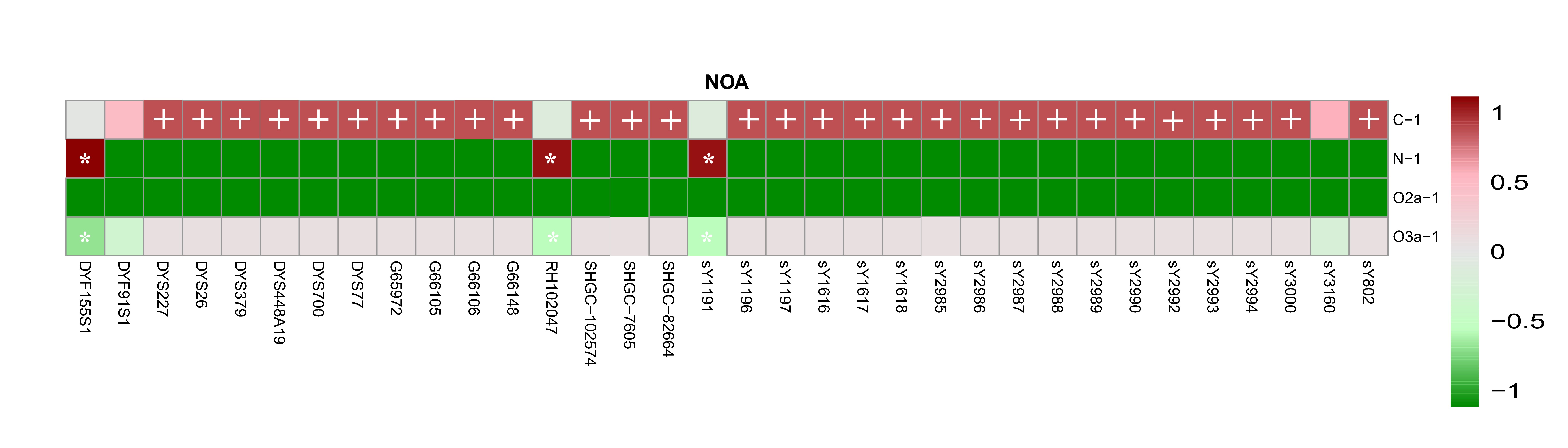


Supplementary Fig. 2


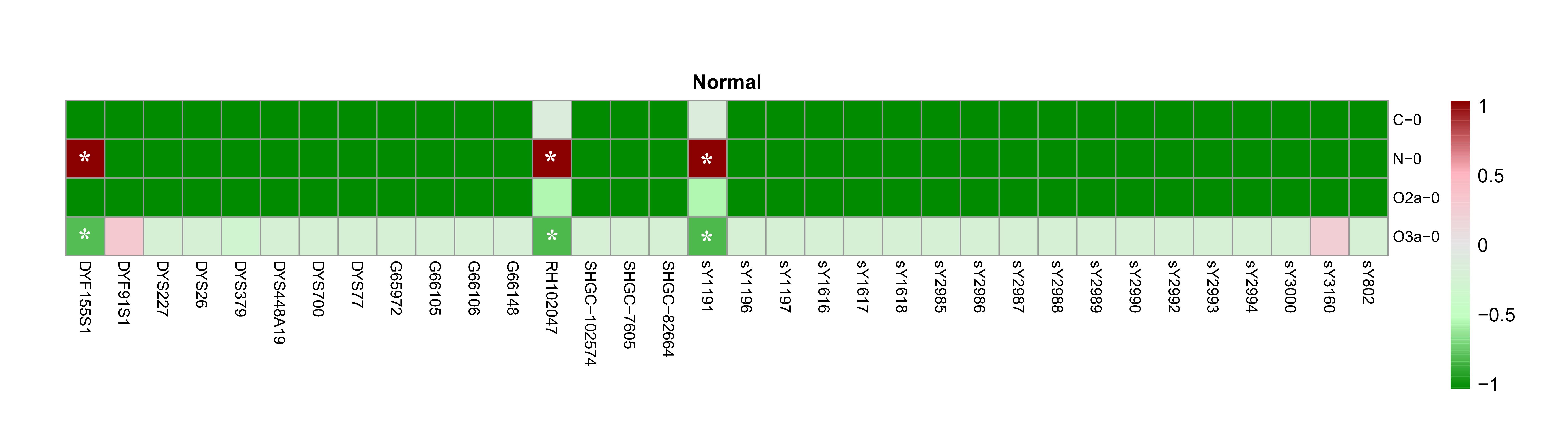


Supplementary Fig. 3


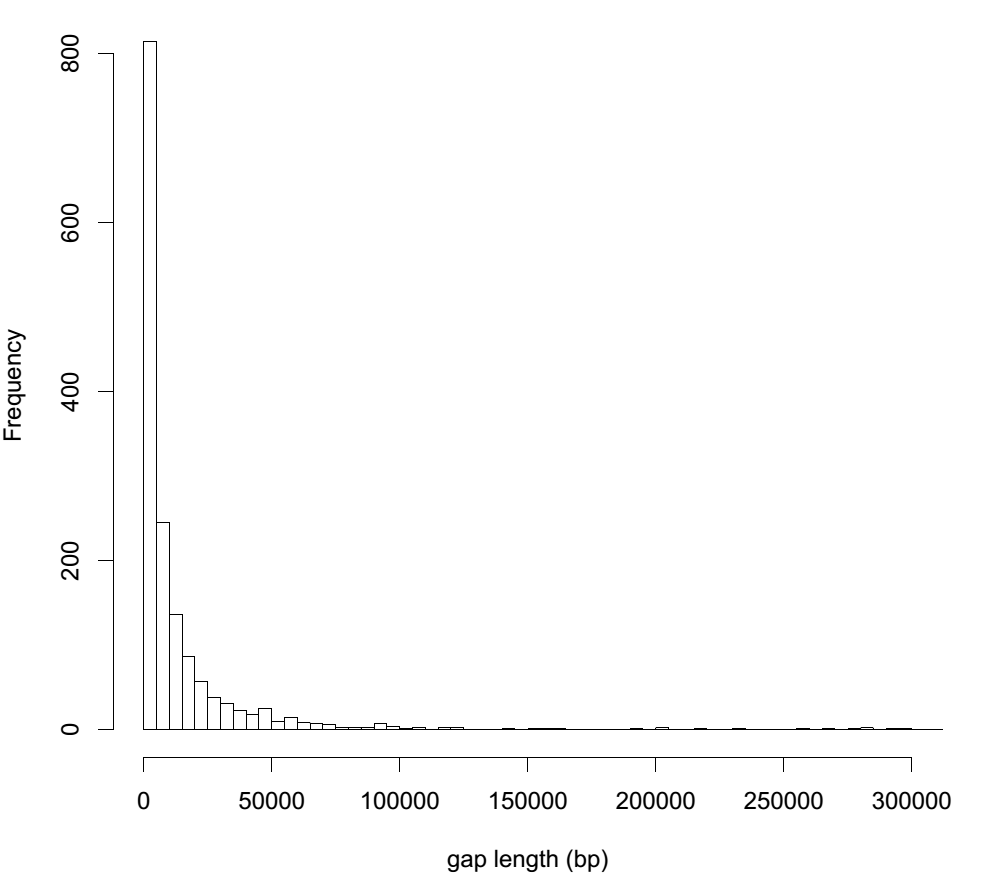


Supplementary Fig. 4
